# Supplementary material for: MetaQTL: a package of new computational methods for the meta-analysis of QTL mapping experiments
Source: BMC Bioinformatics. 2007 Feb 8;8:49. doi: 10.1186/1471-2105-8-49 (PMC1808479; doi:10.1186/1471-2105-8-49)
Supplement: Additional File 7 — MetaQTL Package : jar file and tutorial. This Zip archive contains both the MetaQTL JAR file and the files of the tutorial. [file 1471-2105-8-49-S7.zip › org.inra.metaqtl/doc/org/thalia/bio/class-use/IBioIndividualTree.html]

Uses of Interface org.thalia.bio.IBioIndividualTree


|  |  |  |
| --- | --- | --- |
| **Overview** \| **Package** \| **Class** \| **Use** \| **Tree** \| **Deprecated** \| **Index** \| **Help** | |  |
| PREV   NEXT | **FRAMES**    **NO FRAMES**     **All Classes** |  |


---


## **Uses of Interface org.thalia.bio.IBioIndividualTree**

| Packages that use IBioIndividualTree | |
| --- | --- |
| **org.thalia.bio.entity** |  |

Uses of IBioIndividualTree in org.thalia.bio.entity

| Methods in org.thalia.bio.entity that return IBioIndividualTree | |
| --- | --- |
| `IBioIndividualTree` | `Individual.getPedigree()` |

| Methods in org.thalia.bio.entity with parameters of type IBioIndividualTree | |
| --- | --- |
| `void` | `Individual.setPedigree(IBioIndividualTree pedigree)` |

---


|  |  |  |
| --- | --- | --- |
| **Overview** \| **Package** \| **Class** \| **Use** \| **Tree** \| **Deprecated** \| **Index** \| **Help** | |  |
| PREV   NEXT | **FRAMES**    **NO FRAMES**     **All Classes** |  |


---
